# The Attacking Process in Football: A Taxonomy for Classifying How Teams Create Goal Scoring Opportunities Using a Case Study of Crystal Palace FC

**DOI:** 10.3389/fpsyg.2019.02202

**Published:** 2019-10-16

**Authors:** Jongwon Kim, Nic James, Nimai Parmar, Besim Ali, Goran Vučković

**Affiliations:** ^1^London Sport Institute, School of Science and Technology, Middlesex University, London, United Kingdom; ^2^Fulham Football Club, London, United Kingdom; ^3^Faculty of Sport, University of Ljubljana, Ljubljana, Slovenia

**Keywords:** attacking process, playing patterns, unstable situations, football, taxonomy

## Abstract

**Purpose:**

Whilst some studies have comprehensively described the different features associated with the attacking process in football they have not produced a methodology of practical use for performance enhancement. This study presents a framework of comprehensive and meaningful metrics to objectively describe the attacking process so that useful performance profiles can be produced.

**Methods:**

The attacking process was categorized into three independent situations, no advantage (stable), advantage, and unstable (potential goal scoring opportunity) situations. Operational definitions for each situation enhanced their reliability and validity. English Premier League football matches (*n* = 38) played by Crystal Palace Football Club in the 2017/2018 season were analyzed as an exemplar.

**Results:**

Crystal Palace FC created a median of 53.5 advantage situations (IQR = 16.8) and 23 unstable situations (IQR = 8.8) per match. They frequently utilized wide areas (Median = 21.5, IQR = 9.8) to progress, but only 26.6% resulted in unstable situations (Median = 6.0, IQR = 3.8), the lowest rate compared to the other advantage situations.

**Conclusion:**

This classification framework, when used with contextual factors in a multi-factorial manner, including individual player contributions, will provide practically useful information for applied practice. This approach will help close the so called theory-practice gap and enable academic rigor to inform practical problems.

## Introduction

Football is an invasion sport with the main aim of breaking through an opponent’s defense to score a goal. Since goal scoring is the key to being a successful football team (Wright et al., 2011), many previous notational analysis studies have concentrated on the measurement of scoring related indicators (Hughes and Bartlett, 2002). For example, Reep and Benjamin (1968) identified that 10 shots were needed for one goal and 80% of goals scored from less than 3 passes. Future goal scoring studies considered the impact of: the number of passes in a possession (Hughes and Franks, 2005), pitch area where goals were scored from Yiannakos and Armatas (2006), body part used (Muhamad et al., 2013), set-piece or open play (Muhamad et al., 2013), action prior to a goal (Michailidis et al., 2013) and time period (Armatas et al., 2009) on the number of goals scored. These studies measured the who, when and where goals were scored but neglecting, to some extent, the how but entirely the why.

Match analysis, from a coach’s perspective in the applied world, will invariably focus on the why and how events occurred (Lames and McGarry, 2007) rather than the simple statistics prevalent in the research literature, the so called theory-practice gap (Mackenzie and Cushion, 2013). Mackenzie and Cushion (2013) critically reviewed performance analysis in football over five decades and suggested that a focus on key performance indicators was prevalent, based on availability rather than for developing a deeper understanding of performance. James (2009) also made the point that unless the processes undertaken to achieve outcomes are investigated then meaningful performance improvement information cannot be achieved. This academic perspective is quite different from the approach taken by coaches who plan training sessions following a comprehensive analysis of factors such as the opposition’s strengths and weaknesses and attacking/defending playing patterns (Borrie et al., 2002), referred to as tactical analysis (Garganta, 2009). This process typically involves both the team being coached, and the forthcoming opponents, as it is the interaction between the two teams that coaches try to manipulate. From the theoretical perspective, Hewitt et al. (2016) suggested that identifying playing patterns (referred to as playing style), using more detailed analyses than evident in the literature, would impact training practices, and enable coaches and sport scientists to have a clearer understanding of what teams need to do in order to win. This view strongly advocates the analysis of the “developmental processes” involved prior to a team having goal scoring opportunities. This approach, therefore, requires a systematic breakdown of how teams develop ball possessions into goal scoring opportunities and goals, with the added benefit that this methodology would also enable recurrent patterns to be discerned, allowing the possibility of developing individual team profiles under different playing conditions.

Researchers have suggested that understanding playing patterns could help the development of tactical strategies to improve a team’s performance (James et al., 2002; Tenga et al., 2015). Playing patterns have usually been divided into “possession play” or “direct play” through measuring the number of passes prior to goal (Reep and Benjamin, 1968; Bate, 1988; Hughes and Franks, 2005; Redwood-Brown, 2008) or duration of team possessions (James et al., 2002; Jones et al., 2004; Bloomfield et al., 2005; Lago and Martìn, 2007; Lago, 2009; Lago-Peñas and Dellal, 2010). These studies suggested that playing patterns could be discriminated through a simple data selection process (Fernandez-Navarro et al., 2016) e.g., possession play determined for longer possession durations or number of passes. However, this approach does not allow for possessions which contain elements of different playing patterns. For example, a possession involving multiple passes between defenders in their defensive third of the pitch (generally regarded as possession play) followed by a long pass to an attacker in the attacking third (direct play) would simply be classified as possession play. Thus, this methodology has the potential for failing to classify possession types fully (if multiple possession types were nor classified) or correctly (if one possession type was deemed to supersede another).

Other studies measured multidimensional qualitative variables e.g., direction, type and distance of passes, location where possession started, speed of attack etc., to discriminate playing patterns (Sarmento et al., 2010; Tenga et al., 2010; Tenga and Sigmundstad, 2011; Lago-Ballesteros et al., 2012; Sarmento et al., 2014). Kempe et al. (2014) calculated an index of offensive behaviors (positive values indicated possession play, negative values direct) to characterize playing patterns which included 11 parameters related to passing, direction, speed, accuracy, distance and player involvement. Recently, factor analysis was used to classify team playing style by grouping performance variables perceived to be relevant measures. For example, Fernandez-Navarro et al. (2016) clustered four possession features (direct/possession, cross/no cross, wide/narrow and fast/slow progression) that identified 8 different attacking patterns of play i.e., features that were not mutually exclusive but could present the propensity to utilize a particular attacking pattern. Similarly, Lago-Peñas et al. (2017) measured 20 variables to elicit 5 factors (possession, counter attack, set-piece, regaining ball and losing ball) where values for each factor discriminated how much each team utilized each specific playing pattern. Gomez et al. (2018) extracted 8 factors (ball possession, ending actions, individual challenges, counter attack, set-piece, transitional play, fouling actions and free-kick) and identified changes of team style according to the situational variables match location and team quality.

Although previous papers identified different team playing styles, based on overall match statistics, the authors have typically not distinguished the “how” different attacking procedures evolved e.g., how teams initiate or develop build-up play, progress attacks, create goal scoring opportunities. Some papers have tried to analyze the process of creating goal scoring opportunities by measuring pertinent performance variables such as possession start zone, penultimate action and finishing action (Gonzalez-Rodenas et al., 2019; Mitrotasios et al., 2019). However, these studies simply determined which areas or actions were most prevalent in goal scoring possessions. Kim et al. (2019) suggested that different quality English Premier League (EPL) teams created unstable situations (defined as potential goal scoring opportunities) in different ways. Five specific potential goal scoring situations were identified according to pitch location, game situation or specific action using coach and analyst validated definitions. However, “how” these specific moments in the game arose remains unanswered.

Therefore, the aims of this paper were, (1) to establish a taxonomy of the different ways in which potential goal scoring opportunities (unstable situations) arise and (2) to provide a framework for identifying team profiles for attacking patterns of play. This will provide a rigorous methodology for players and coaches to collect information pertinent to identifying an opponent’s attacking patterns. Additional information regarding individual player names (not used in this methodology) would thus generate the type of information appropriate to plan training sessions and game plans for upcoming matches.

## Materials and Methods

### Sample

All the league matches (*n* = 38) played by Crystal Palace Football Club in the EPL in the 2017/2018 season were selected. All data, including video footage of the all matches, was officially provided by the football club. Ethical approval for the study was provided by the sports science sub-committee of Middlesex University’s ethics committee in accordance with the 1964 Helsinki declaration.

### Creating a Taxonomy for the Process of Creating Unstable Situations

**FIGURE 1:**
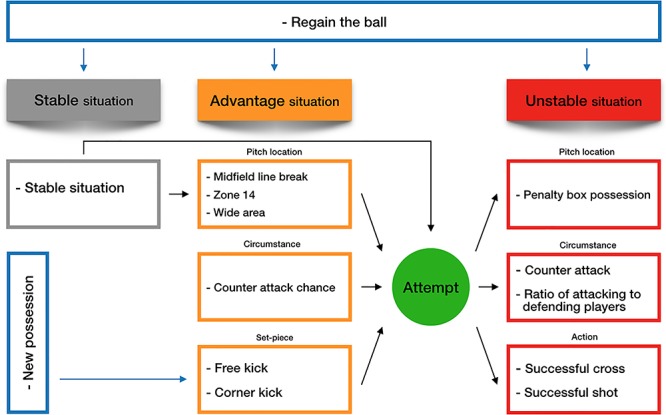
A framework for categorising the attacking process in football.

This study describes the attacking process by differentiating stable, advantage and unstable situations (Figure 1). Each team possession could start by regaining the ball from the opponent, in any of these three situations or with a new possession i.e., a set piece (lines in Figure 1 indicate the start and progression of possessions).

Operational definitions were devised for each situation to enhance their reliability and validity. A stable situation was defined as a situation in which neither team had a clear advantage. This occurred when a team had possession of the ball in their middle or defensive third of the pitch and the opponents were in their normal positions with their midfield and defenders goal side of the ball.

**FIGURE 2:**
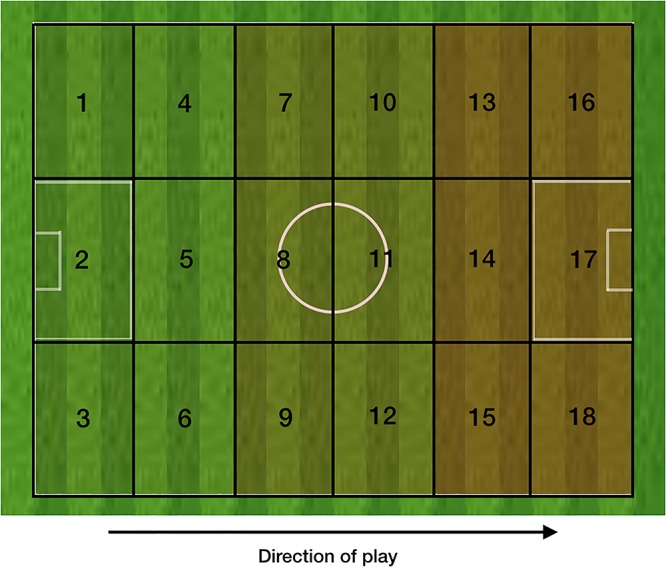
The pitch of play divided into 18 zones.

**FIGURE 3:**
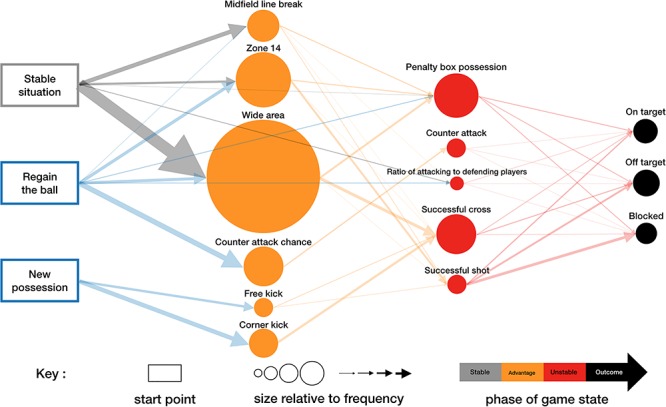
The attacking process network of Crystal Palace Football Club in the 2017/2018 season.

The advantage situation was deemed to occur when the game state changed to one where the possibility of an unstable situation arising became clear. These situations arose when (1) a team in possession broke the opposition team’s midfield line i.e., had possession between their midfield and defensive lines; (2) a team had possession in zone 14 (Figure 2); (3) a team had possession in a wide area of the final third of the pitch with the opportunity to pass, cross or dribble into the penalty box or shoot directly at the goal; (4) a team regained the ball and had the opportunity to counter attack; (5) free kick in position where a shot or cross was possible; (6) corner kick.

Unstable situations were previously defined by Kim et al. (2019), who validated the five specific situations used here (Figure 1). Penalty kicks were excluded from both papers because penalties are the consequence of an attack and the kick deemed a new possession.

### Procedure

All matches were viewed and coded in SportsCode Elite v10.3.36, to enable time stamps for each advantage state and when unstable situations arose (see also Kim et al., 2019). Apple Movist v1.3.6 was also used to facilitate coding due to ease of video manipulation.

On some occasions, a team in possession of the ball could be described in more than one category of advantage situation during a single possession. For example, if a team in possession in zone 14 switched the ball into a wide area, the two advantage situations were coded separately so that each specific situation was recorded. Similarly, different unstable situations could occur during a single possession. In this scenario, only the first unstable situation was coded because the aim of the study was to identify the moment the game state changed (stable to unstable) e.g., a counter attack could result in a penalty box possession situation but the latter was deemed irrelevant as there was no game state change between the counter attack and the penalty box possession. This could, however, be on interest to future analyses.

### Reliability

Intra- and inter-observer tests were performed to determine whether the advantage situations (*n* = 6), unstable situations (*n* = 5) and outcomes (*n* = 3) were reliably categorized (James et al., 2007). The researcher (intra-, over four weeks after the first coding to nullify memory effects) and an independent experimenter (inter-, who was trained for each operational definition) re-coded three randomly selected matches using the same post-event coding procedure as outlined above. Advantage situations had high Kappa values for intra- (0.97, *n* = 362 comparisons) and inter- experimenter (0.86, *n* = 372). Discrepancies tended to arise when an experimenter missed an event especially wide area chances (intra = 2 and inter = 12). Also, Unstable situations had high Kappa values for intra- (0.94, *n* = 138) and inter- (0.87, *n* = 146). Discrepancies tended to arise when an experimenter failed to distinguish counter attacks (intra = 3 and inter = 8). Outcome had the same high Kappa value for both inter- and intra- (0.96, *n* = 76).

### Statistical Analysis

All data were analyzed in IBM SPSS 25.0. Descriptive statistics were performed to provide median and interquartile range values for advantage, unstable situations and outcomes as variables were skewed. A Kruskal–Wallis *H* test was used to determine statistical differences for each situation and Mann–Whitney *U* test used to compare playing at home and away. The level of significance set at *p* < 0.05.

## Results

Crystal Palace football club created a median of 53.5 advantage situations (IQR = 16.8), 40 attempts (IQR = 11.3), 23 unstable situations (IQR = 8.8), 12 shots (IQR = 6.8) and 1 goal (IQR = 2) per match (Figure 3). Most unstable situations developed from advantage situations (Median = 20.5, IQR = 7.8) with a few from possession regains in unstable situations (Median = 2.5, IQR = 2.8) and from stable situations which did not involve an intermediary advantage situation (Median = 1, IQR = 1.8) i.e., a long ball.

**FIGURE 4:**
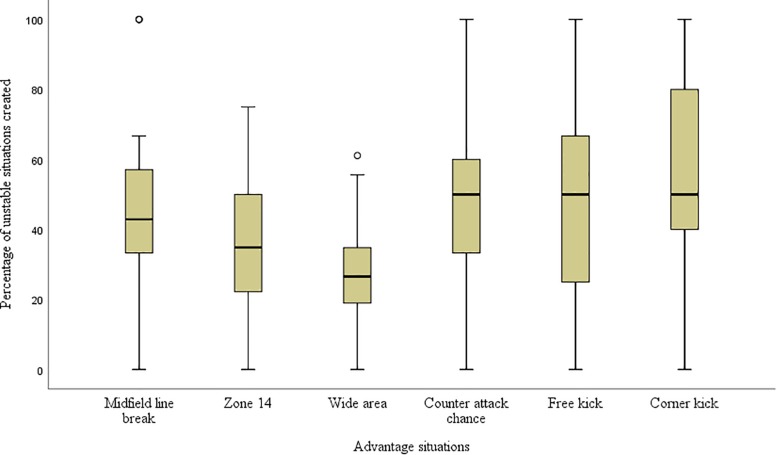
Percentage of unstable situations created from advantage situations.

Crystal Palace created 21.5 wide area chances (IQR = 9.8) per match, 41.4% of all advantage situations, which was significantly higher (χ^2^ = 88.63, *p* < 0.05) than the other five advantage situations (midfield line break- Median = 5.5, IQR = 5.0, zone 14- Median = 10.0, IQR = 5.0, counter attack chance- Median = 7.0, IQR = 5.0, free kick- Median = 3.0, IQR = 2.0, corner kick- Median = 5.0, IQR = 4.0). However, only 26.6% of wide area chances resulted in unstable situations, the lowest rate (χ^2^ = 190.0, *p* < 0.05) compared to the others (Figure 4).

A total of 79.9% unstable situations occurred from open play (Median = 18.5, IQR = 7.8) and 20.1% from set piece (Median = 4.0, IQR = 3.0). Unstable situations were most likely to be penalty box possessions (Median = 8.0, IQR = 5.0) or successful crosses (Median = 7.0, IQR = 4.5), accounting for 63.5% of all unstable situations (χ^2^ = 54.0, *p* < 0.05, Figure 3). Penalty box possessions occurred from midfield line break chances (Median = 1.0, IQR = 2.0), zone 14 chances (Median = 1.0, IQR = 1.0), wide area chances (Median = 2.5, IQR = 2.0) and regaining the ball directly in an unstable situation (Median = 1.0, IQR = 2.0).

Shots were most likely to occur from successful crosses (Median = 3.0, IQR = 2.5) and successful shot (Median = 3.0, IQR = 3.0) situations (χ^2^ = 56.71, *p* < 0.05) compared to the other unstable situations whilst shots on target occurred most frequently from successful crosses (Median = 1.0, IQR = 1.0) and penalty box possessions (Median = 1.0, IQR = 2.0) (χ^2^ = 16.78, *p* < 0.05). However, in terms of the rate of creating shots, the ratio of attacking to defending players was the most likely situation to result in a shot (48.8%, χ^2^ = 57, *p* < 0.05), a shot on target (22.1%, χ^2^ = 16.78, *p* < 0.05) and a goal (5.8%, χ^2^ = 15.8, *p* < 0.05).

There were no significant differences for total advantage situations, unstable situations and shots (all *p* > 0.05, Table 1) between playing at home and away. In detail, however, they created more penalty box possession unstable situations (*p* < 0.05) when playing at home (Median = 10.0, IQR = 5.5) than away (Median = 7.0, IQR = 4.0).

**TABLE 1:** Frequency of specific advantage and unstable situations by match location.

**Advantage situation**						**Unstable situation**					
	**Home**		**Away**		***p***		**Home**		**Away**		***p***
	**Median**	**IQR**	**Median**	**IQR**			**Median**	**IQR**	**Median**	**IQR**	
MLB	5.0	4.0	6.0	5.5	0.67	PBP	10.0	5.5	7.0	4.0	0.02
Z14	12.0	6.0	9.0	4.0	0.05	CA	3.0	4.0	3.0	2.5	0.98
WA	26.0	10.5	20.0	6.0	0.75	RAD	2.0	6.0	2.0	1.5	0.64
CAC	7.0	6.0	7.0	4.0	0.15	SC	7.0	4.0	6.0	4.0	0.42
FK	3.0	3.0	3.0	1.5	0.18	SS	3.0	1.5	3.0	2.5	0.77
CK	5.0	2.5	5.0	4.0	0.95						
Total	59.0	14.0	51.0	12.5	0.10	Total	25.0	7.0	22.0	7.0	0.10

*MLB = midfield line break, Z14 = zone 14, WA = wide area, CAC = counter attack chance, FK = free kick, CK = corner kick, PBP = penalty box possession, CA = counter attack, RAD = ratio of attacking to defending players, SC = successful cross, SS = successful shot.*

## Discussion

The identification of a team’s playing pattern is highly likely to be beneficial to coaches and sport scientists as this would impact training methodologies as a consequence of having a clear understanding of what teams need to do in order to win (Hewitt et al., 2016). The academic literature, however, has often considered playing pattern as simply “direct play” or “possession play,” determined by simplistic measures such as the number of passes (e.g., Reep and Benjamin, 1968; Bate, 1988) or duration of team possessions (e.g., Jones et al., 2004; Hughes and Franks, 2005). This classification has clear face validity, given that the terms are ubiquitous in the football media, but offer little insight to applied practice whose goal is performance improvement. This limitation has prompted more recent research to consider multidimensional qualitative variables (e.g., offensive behaviors, Kempe et al., 2014; and factor analysis, Fernandez-Navarro et al., 2016; Lago-Peñas et al., 2017; Gomez et al., 2018). Gonzalez-Rodenas et al. (2019) presented specific actions e.g., penultimate or finishing action and subspaces i.e., areas of the pitch involved in the play, prior to goals being scored. However, no information was provided regarding how teams developed their attacks e.g., midfield line breaks or counter attacks. Similarly, Mitrotasios et al. (2019) presented attacking categories i.e., counter, combinative, fast and direct attacks, but did not consider any further details such as pitch locations, players involved etc. These studies comprehensively described the different features associated with the attacking process but failed to produce a methodology of practical use for performance enhancement. It was this limitation that prompted this study i.e., the development of a classification framework of the attacking process in football, with the aim of providing a suitable methodology for applied practice.

Kim et al. (2019) presented five specific situations that were described as unstable situations, more importantly defined as potential goal scoring opportunities. Of interest here was “how” one team achieved these in different situations. An analysis of all 38 matches in the 2017/2018 EPL suggested that the attacking process can be encapsulated by three different game situations, stable, advantage and unstable. These situations did not occur for every possession and the transition between situations was not uniform. Indeed, possession could originate in any of the situations with the way a team plays (playing pattern) likely to determine the frequency of each situation. For example, a team that employs the high press frequently is likely to win possession in an unstable situation more often than a team that does not.

In this study, 79.9% of unstable situations occurred in open play situations, which was similar to the occurrence of penultimate actions leading to goals during open play (75.9%; Gonzalez-Rodenas et al., 2019). Crystal Palace were shown to frequently utilize the wide areas to progress their attacks which resulted in their goal scoring opportunities as a consequence of penalty box possessions and successful crosses. The corner kick was shown to be their most effective method of creating an unstable situation. It is widely perceived that Crystal Palace’s best players operate in the attacking wide areas i.e., wingers, Wilfred Zaha and Andros Townsend. It was thus not surprising that these analyses showed the prevalence of attacks from wide areas. Similarly, fullbacks Wan-Bissaka and Patrick van Aanholt are recognized as the players who make passes to the wingers and support their play in the wide area. This paper did not include player names as the purpose was to generate a rigorous methodology rather than a specific analysis of a team. However, names of players would be utilized by teams adopting this approach given their requirement of producing a tactical game plan to defeat a future opponent. The emphasis of Crystal Palace’s attacking play using the wide areas supports the notion that they do not have players like to hold onto the ball in midfield areas and build up play using good passes, hence low midfield line breaks and low zone 14 possessions.

Since this research developed a previous study by Kim et al. (2019) no record was made of unstable situations that occurred subsequent to an initial one occurring during a single team possession. This meant that an accurate portrayal of all unstable situations was not possible. However, this extra information relates to how unstable situations sometimes develop and may provide additional information of value in the future. Similarly, the time during which events took place was not recorded. Temporal information may elucidate specific patterns e.g., Manchester City are well known for slow build up play i.e., the average time of their possessions in stable situations would be very different to a team like Leicester City who tend to focus on quick counter attacks. Time has also been shown to be useful in t-pattern analysis (Borrie et al., 2002) and would be a useful tool for further exploring this type of data. Other factors such as the number of passes, forward passes etc. were also omitted from this study, some of which have been used to discriminate playing patterns e.g., distance of passes (Tenga et al., 2010). Long passes are generally associated with direct play where defenders or midfielders pass to forward near the opponent’s defensive line. In this study these passes were classified as either counter attacks (regain the ball in advantage situation) or when situations changed from stable to unstable but bypassed the advantage situation. Hence, the playing patterns generally referred to as “possession” would typically involve transition from stable to midfield line breaks/zone 14/wide area to penalty box possessions. In contrast direct play would miss out some of these situations either by involving no stable situation or missing out the advantage situation.

This study analyzed all matches from a season without considering well-known factors likely to influence performance. For example, match status, whether a team is winning, drawing or losing at the time and opponent quality have all been shown to influence performance. A simple analysis of the effect of match venue showed that Crystal Palace produced slightly more penalty box possession at home compared to away but this did not consider the other factors of importance. In future studies, these factors need to be investigated in a multi-factorial manner e.g., how does a team play when losing against a top rated opponent playing away. This classification framework also needs to be expanded to include individual player contributions if practically useful information is to be gained. Whilst academic literature tends to gravitate toward large data sets and statistical significance the usefulness of such an approach has been questioned for practically useful insights (Mackenzie and Cushion, 2013).

## Conclusion

A novel methodology for classifying the attacking process in football has been presented with a view to providing a scientifically valid approach for use in the applied world. However, for this framework to be of practical benefit, future analyses need to consider contextual information in a multi-factorial manner. In this way teams can analyze their future opponents to determine how they create goal scoring opportunities during different scenarios, such as when their main striker is not playing.

## Data Availability Statement

All datasets generated for this study are included in the manuscript/supplementary files.

## Author Contributions

NP and BA assisted in reliability testing, data collection, and error checking. JK, NJ, and GV designed the study, conducted the analysis, interpreted the data, and wrote the manuscript. All authors read and approved the final manuscript.

## Conflict of Interest

BA was employed by company Fulham Football Club.

The remaining authors declare that the research was conducted in the absence of any commercial or financial relationships that could be construed as a potential conflict of interest.
